# Antimicrobial activity, toxicity and anti-inflammatory potential of methanolic extracts of four ethnomedicinal plant species from Punjab, Pakistan

**DOI:** 10.1186/s12906-017-1815-z

**Published:** 2017-06-08

**Authors:** Rabia Naz, Hafsa Ayub, Sajid Nawaz, Zia Ul Islam, Tayyaba Yasmin, Asghari Bano, Abdul Wakeel, Saqib Zia, Thomas H. Roberts

**Affiliations:** 1 Department of Biosciences, COMSATS Institute of Information Technology, Islamabad, Pakistan; 2 Department of Sociology and Anthropology, PMAS University of Arid Agriculture, Rawalpindi, Pakistan; 3 Department of Plant Sciences, Quaid-i-Azam University, Isamabad, Pakistan; 4 Department of Mathematics, COMSATS Institute of Information Technology, Islamabad, Pakistan; 5 Plant Breeding Institute, Sydney Institute of Agriculture, University of Sydney, Sydney, NSW 2006 Australia

**Keywords:** Plant extracts, Phytochemicals, TLC, Antimicrobials, Antioxidants, Cytotoxicity, Anti-inflammatory, Anti-proteinase, Chowk Azam

## Abstract

**Background:**

The plant species *Aristolochia indica* (AI)*, Melilotus indicus* (MI), *Tribulus terrestris* (TT) and *Cuscuta pedicellata* (CP) are widely used in folk medicine in the villages around Chowk Azam, South Punjab, Pakistan. The aim of this study was to evaluate the antioxidant activity, phytochemical composition, and the antibacterial, antifungal, cytotoxic and anti-inflammatory potential of the four medicinal plants listed above.

For CP stem, this study represents (to the best of our knowledge) the first time phytochemicals have been identified and the antioxidant and anti-inflammatory potential determined.

**Methods:**

Phytochemicals were analyzed through chemical tests, thin layer chromatography (TLC) and spectrophotometric methods. Antioxidant activities (DPPH and H_2_O_2_) were also determined through spectrophotometric methods. Extracts were evaluated for antibacterial potential via the agar well diffusion method against *Staphylococcus aureus, Pseudomonas aeruginosa*, *Klebsiella pneumonia* and *Acinetobacter baumannii*. The minimal inhibitory concentration (MIC) and minimal bactericidal concentration (MBC) were determined by the microdilution method. Antifungal activities were tested using the agar tube dilution method against three species: *Aspergillus fumigatus, Aspergillus flavus* and *Rhizopus oryzae*. The cytotoxic potential of the plant extracts was checked using the brine shrimp assay. *In vitro* anti-inflammatory activity of the selected plant extracts was evaluated using albumin denaturation, membrane stabilization and proteinase inhibitory assays.

**Results:**

Of all the methanolic extracts tested, those from CP (stem) and TTF (*T. terrestris* fruit) had the highest phenolic, flavonoid and flavonol contents (497±4 mg GAE/g, 385±8 mg QE/g and 139±4 mg QE/g; 426±5 mg GAE/g, 371±8 mg QE/g and 138±6 mg QE/g, respectively) and also exhibited strong antioxidant potential in scavenging DPPH and hydrogen peroxide (IC_50_ values; 20±1 and 18±0.7 μg/mL; 92±2 and 26±2 μg/mL, respectively). CP, TTF and TTL (*T. terrestris* leaf) extracts substantially inhibited the growth of the bacteria *A. baumannii, S. aureus,* and *K. pneumonia* and also exhibited the highest antifungal potential. The ranking of the plant extracts for cytotoxicity was TTF > TTL > AI > CP > MI, while the ranking for *in vitro* anti-inflammatory potential at a concentration of 200 μg/mL of the selected plant extracts was CP > TTL, TTF > AI > MI. The lowest IC_50_ (28 μg/mL) observed in the albumin denaturation assay was for CP. Positive correlations were observed between total phenolics, antioxidants, antibacterial, antifungal and anti-inflammatory potential of the selected plant extracts, indicating a significant contribution of phenolic compounds in the plant extracts to these activities.

**Conclusions:**

This study revealed the strong antimicrobial, antioxidant, cytotoxic and anti-inflammatory potential of the plant species CP and TT used in folk medicine.

## Background

Tissues of many plants species contain secondary metabolites with the potential to combat disease-causing micro-organisms. These compounds include glycosides, saponins, flavonoids, steroids, tannins, alkaloids and terpenes [1]. Extracts of different plant organs, including roots, leaves, bark, flowers, fruits and seeds, may contain distinct phytochemicals with activity against bacterial or fungal pathogens [2]. In folk medicine, a single plant species is often used to treat more than one type of disease or infection [3]. Extracts of plants with a history of traditional use should be tested using modern methods for activities against human pathogens, with the aim of discovering potential new drugs.

Inflammatory diseases comprising various kinds of rheumatism are common all over the world [4]. Although rheumatism is the oldest known human disease, limited progress has been made for its permanent treatment. Non-steroidal anti-inflammatory drugs (NSAIDs) are being used to cure and control inflammation, fever and pain. However, their use has not been therapeutically efficacious in all types of inflammations. Furthermore, the use of NSAIDs can cause adverse side-effects leading to hemorrhage and ulcers [5].

Pakistan has a rich diversity of medicinal and aromatic plants due to its unique phytogeography with diverse climatic conditions. The Chowk Azam town to the east of district Layyah lies in the sub-tropical continental plain of Pakistan, which features sandy soil. The climate is extremely hot in summer, while in winter temperatures can drop to 0 °C. People living in the villages around Chowk Azam rely mainly on medicinal plants to treat their minor diseases, and in some cases major diseases like cancer and hepatitis [6].

Four medicinal plants were studied here: *Aristolochia indica* (Aristolochiaceae), a creeper plant, perennial herb or shrub [7]; *Cuscuta pedicellata* (Convolvulaceae), a holoparasitic annual that is usually observed as dense tangles of fine, yellow-orange, heavily-branched stems in the foliage of host plants [8]; *Melilotus indicus* (Fabaceae), an annual noxious weed of crop fields, gardens, orchards and canal banks [9] and *Tribulus terrestris* (Zygophyllaceae), which features a stem that remains close to the ground, covered with lint [10]. These plants are used by the local community in the villages of Chowk Azam in the district Layyah, Pakistan, for the treatment of various skin infections/irritations, poisonous bites, inflammation and some other infectious diseases (Table 1).

**Table 1:** Profile of ethnomedicinal use of selected medicinal plants, botanical/English/local name used, route of administration and plant extract yield obtained in the present study

Sample no.	Botanical name, family	English name/Local name	Parts used	Ethnomedicinal use	Route of administration	Yield (%)
1	*Aristolochia indica* L., Aristolochiaceae	Birthwort,wort killer/ Hukka-bel	Leaves	To cure poisonous bite, inflammations, reduce itching, leprocy, gastric stimulant, diarrhoea, intermittent fever, cough	Topical, Oral	31
2	*Cuscuta pedicellata* Ledeb, Convolvulaceae	Clover dodder/ Loot booti (Saraiki) Akash-bail/Amar-bail (Urdu)	Stem	stomachache, to cure wounds, cuts, used as purgative, anti-inflammatory and to treat high blood pressure	Oral, Topical	17
3	*Melilotus indicus* L., Fabaceae	Yellow melilot (English) Sweet clover/ Sinjee	Leaves	To cure inflammations and skin irritation, astringent, anticoagulant, laxative	Oral, Topical	29
4	*Tribulus terresteris* L., Zygophyllaceae	Puncture clover/ Bhakra, Gokhru	Leaves	anti-inflammatory, lithotriptic, diuretic, general tonic		37
5	*Tribulus terresteris*		Fruit	anti-inflammatory, effective in most of Gynecological and genitourinary disorders, Gonorrhoea and to treat abdominal distension	Topical, Oral	33

Key techniques employed in this study include the brine shrimp lethality and cytotoxicity assays, which are known to detect a wide range of bioactivities in plant crude extracts. The lethality potential of plant extracts to brine shrimp is an effective method of prescreening prior to conducting more elaborate antitumor and cytotoxicity assays. Numerous studies have reported the use of the brine shrimp assay to screen plant extracts for activities against fungi, arthropod pests (including insect larvae) and molluscs, as well as their anticancer and cytotoxic potential. The cytotoxicity brine shrimp assay has also been used successfully as a step in the identification of antineoplastic, cytotoxic, antimalarial, antifeedant and insecticidal compounds from many plants [11].

The aim of this study was to determine whether the selected plant extracts can control the growth of pathogenic microorganisms. We focused on the phytochemical, antioxidant, antimicrobial, cytotoxicity and anti-inflammatory properties of the four medicinal plant species.

## Methods

### Chemicals and collection of plant materials

Methanol, Folin–Ciocalteu reagent, 2,2-diphenyl-1-picrylhydrazyl (DPPH), quercetin, rutin, gallic acid, aluminum chloride, potassium acetate, sodium acetate, ascorbic acid, hydrogen peroxide, phosphate buffer, nutrient broth, dimethyl sulfoxide (DMSO), potato dextrose agar, terbinafine, streptomycin, bovine serum albumin (BSA), casein, perchloric acid, aspirin, Tris-HCl, and trypsin were purchased from Sigma-Aldrich. All reagents and chemicals were of analytical grade.

Samples of the plant species *Aristolochia indica* (AI)*, Cuscuta pedicellata* (CP) (stems)*, Melilotus indicus* (MI) and *Tribulus terresteris* fruit (TTF) and leaf extracts (TTL) were collected from Chowk Azam, Layyah District, Punjab, Pakistan. The plants were identified by Prof. Dr. Mir Ajab Khan, Department of Botany, Quaid-i-Azam University. The plant species were selected on the basis of reports obtained from traditional herbalists and local people that extracts are used mainly to treat various infectious diseases. The scientific, English and local names, the parts of the plants extracted traditionally, their ethnomedicinal uses, route of administration and extract yields for the four plant species are described in Table 1.

### Preparation of plant extracts

Plant leaves, stems and fruits of the selected plant species were washed thoroughly with distilled water and shade-dried for 3 d at room temperature. The dried leaves, stems and fruits were uniformly ground using an electric grinder. The powdered plant material (250 g) was extracted for 4 d in 1 L 100% methanol [12]. The separated extracts were then filtered through Whatman No. 1 filter paper and the methanol filtrate evaporated to dryness using a rotary evaporator at room temperature (30 °C). The thick extracted mass was then dried at room temperature, and the dried extract stored in an air-tight container at 4 °C until further use.

### Determination of plant extract yield (%)

Yield percentage (*w*/w) from the dried extracts was calculated as:

$$ \mathrm{Yield}\ \left(\%\right)=\left(\mathrm{W}1\ast 100\right)/\mathrm{W}2 $$

where W1 is the dry weight of extract after evaporating the solvent and W2 is the weight of the soaked plant powder.

### Preliminary phytochemical screening

Phytochemical screening of the selected plant extracts was performed to detect the presence of phytochemical constituents, including saponins, terpenoids [12], anthraquinones, phlobatannins [13], flavonoids and phenolic compounds [14].

### Thin layer chromatography

The methanolic plant extracts (10 μL) were applied on pre-coated TLC plates using capillary tubes and air dried. The TLC plates were developed in a chamber using chloroform: methanol (5:1) as the mobile phase and observed under UV light (254 nm). Caffeic acid, quercetin, rutin, *trans*-cinnamic acid and salicylic acid were used as standards.

The mobility of the samples was expressed as retention factor (R*f*) as calculated using the following formula:

$$ \mathrm{R} f=\frac{\mathrm{Distance}\ \mathrm{travelled}\ \mathrm{by}\ \mathrm{the}\ \mathrm{solute}\ \left(\mathrm{cm}\right)}{\mathrm{Distance}\ \mathrm{travelled}\ \mathrm{by}\ \mathrm{the}\ \mathrm{solvent}\ \left(\mathrm{cm}\right)} $$

### Quantitative analysis of phytochemicals

#### Total phenolic content

Total phenolic content was analyzed using the Folin–Ciocalteu colorimetric method [15] with some modifications. An aliquot of 0.3 mL of the plant extract was mixed with Folin-Ciocalteu phenol reagent (2.25 mL). After 5 min, 6% sodium carbonate (2.25 mL) was added and the mixture was allowed to stand at room temperature for 90 min. The absorbance of the mixture was measured at 725 nm in a spectrophotometer (HITACHI Model: U-1100 573 × 415). A calibration curve for gallic acid in the range 20–80 μg/mL was prepared in the same manner. Results were expressed as mg gallic acid equivalent (GAE) per gram extract.

#### Total flavonoid content

Total flavonoid content was determined using the aluminum chloride colorimetric method [16, 17] with some modifications. A calibration curve for quercetin in the range 20–80 μg/mL was prepared. Plant extract (0.5 mL) and standard (0.5 mL) were placed in separate test tubes and 10% aluminum chloride (0.1 mL), 1 M potassium acetate (0.1 mL), 80% methanol (1.5 mL) and distilled water (2.8 mL) added and mixed. A blank was prepared in the same manner but 0.5 mL of distilled water was used instead of the sample or standard. All tubes were incubated at room temperature for 30 min and the absorbance was read at 415 nm. The concentration of flavonoid was expressed as mg quercetin equivalent (QE) per gram extract. Each plant extract was made in triplicate.

#### Total flavonol content

Total flavonol content was determined following the aluminum chloride colorimetric method [18, 19] with some modifications. A calibration curve for quercetin in the range 20–80 μg/mL was prepared., Extract (1 mL) and standard (1 mL) were placed in separate test tubes and 2% aluminum chloride (1 mL), 5% sodium acetate (3 mL) added and mixed. The mixture was then centrifuged at 3000 rpm for 20 min to obtain a clear solution. The absorbance was read at 440 nm and the results expressed as mg quercetin equivalent (QE) per gram of extract. Each plant extract was prepared in triplicate.

### DPPH radical scavenging activity

The free radical scavenging activity of the selected plant extracts was measured in vitro via the 2, 2′- diphenyl-1-picrylhydrazyl (DPPH) assay [20, 21]. The reaction mixture (3 mL) consisted of 1 mL DPPH (0.3 mM) in methanol, 1 mL extract and 1 mL methanol. The radical scavenging activity of the samples at various concentrations (25–200 μg/mL) was measured. The reaction mixture was shaken well and incubated in the dark for 10 min at room temperature. Absorbance was read at 517 nm. The control was prepared as above but without any plant sample. Ascorbic acid [22] and rutin [23] were used as positive controls.

Scavenging activity was estimated based on the percentage of DPPH radical scavenged according to the following equation:

$$ \mathrm{Scavenging}\kern0.35em \mathrm{effect}\%=\left[\left(\mathrm{control}\kern0.35em \mathrm{absorbance}{\textstyle \hbox{--}}\mathrm{sample}\kern0.35em \mathrm{absorbance}\right)/\left(\mathrm{control}\kern0.35em \mathrm{absorbance}\right)\right]\times 100 $$

### Hydrogen peroxide radical (H_2_O_2_) scavenging assay

The ability of plant extracts to scavenge hydrogen peroxide was determined according to the method of Ruch et al. (1989) [24]. A solution of hydrogen peroxide (2 mM) was prepared in phosphate buffer (50 mM, pH 7.4). Plant extracts (25–200 μg powder/mL) were prepared in distilled water, and aliqots (0.1 mL) transferred into vials and their volumes made up to 0.4 mL with 50 mM phosphate buffer (pH 7.4). After addition of 0.6 mL hydrogen peroxide solution, tubes were vortexed and the absorbance was determined at 230 nm after 10 min against a blank solution containing phosphate buffer without hydrogen peroxide. Ascorbic acid [25] and rutin were used as positive controls.

The ability of the extract to scavenge hydrogen peroxide was calculated using the following equation:

$$ \%\mathrm{Scavenged}\kern0.35em \left[{\mathrm{H}}_2{\mathrm{O}}_2\right]=\left[\left({\mathrm{A}}_{\mathrm{C}}{\textstyle \hbox{--} }{\mathrm{A}}_{\mathrm{S}}\right)/{\mathrm{A}}_{\mathrm{C}}\right]\times 100 $$

where A_C_ and A_S_ are the absorbance of the control and sample, respectively.

### Pathogenic bacterial and fungal strains used

#### Bacteria

The bacterial pathogens used were *Acinetobacter baumannii* (ATCC 17978), *Staphylococcus aureus* (ATCC 6538)*, Pseudomonas aeruginosa* (ATCC 7221) and *Klebsiella pneumoneae* (ATCC 6059), which were obtained from the Department of Microbiology, Quaid-i-Azam University, Islamabad, Pakistan.

#### Fungi

The fungal pathogens *Aspergillus flavus* (FCBP-PTF-1265) and *Aspergillus fumigatus* (FCBP-MF-923) were obtained from the First Fungal Culture Bank of Pakistan (FCBP), University of the Punjab, Pakistan. *Rhizopus oryzeae* (ATCC 11886 (AY 803930)) was obtained from the Department of Microbiology, Quaid-i-Azam University, Islamabad.

The bacterial isolates were first sub-cultured in a nutrient broth (Sigma) and incubated at 37 °C for 18 h. The fungal isolates were sub-cultured on potato dextrose agar (PDA) (Merck) for 7 d at 25 °C.

### Positive and negative controls

*Streptomycin* (30 μg/mL) and terbinafine (1 mg/mL) were used as positive controls for the antibacterial and antifungal tests, respectively. DMSO was used as negative control for the antibacterial and antifungal analyses.

### Assay for antibacterial activity

Antibacterial activity of the methanol extracts of the selected plant species was determined using the agar well diffusion method [26]. Petri plates were prepared by pouring 75 mL of seeded MH agar and allowing the agar to solidify. Freshly prepared bacterial inoculum was evenly spread using a sterile cotton swab on the entire agar surface. A hole was then punched with a sterile cork borer (6 mm) and 100 μL of each crude extract was poured into the well. Petri plates were then allowed to stand at room temperature for 1 h and incubated at 37 °C overnight. Controls were run in parallel whereby solvent was used to fill the well. The plates were observed for zones of inhibition after 24 h and the results compared with those of the positive control, streptomycin (30 μg/mL).

### Determination of minimum inhibitory concentration (MIC) and minimum bactericidal concentration (MBC)

The determination of MIC of the methanolic extracts of the selected plant species was carried out by the microdilution method [27] using nutrient broth. Plant extracts were dissolved in 10% DMSO and two-fold dilutions were prepared with culture broth. Each test sample and growth control (containing broth and DMSO, without plant extract/antimicrobial substance) was inoculated with 10 μL of bacterial suspension containing 5 × 10^6^ CFU/mL. A 10-μL solution of resazurin (270 mg resazurin tablet dissolved in 40 mL of sterile water) was also added to each sample and incubated for 24 h at 37 °C. Bacterial growth was detected by reading absorbance at 500 nm. Bacterial growth was indicated by a color change from purple to pink or colorless (assessed visually). MIC was defined as the lowest plant extract concentration at which the color changed, or the highest dilution that completely inhibited bacterial growth. The test dilutions were further sub-cultured on fresh solid media and incubated for 18 h to determine the MBC values. The lowest plant extract concentration (highest dilution) that killed all the bacteria was defined as MBC. Experiments were carried out in triplicate to test each dilution for each bacterial strain to determine MIC and MBC values.

### Assay for antifungal activity

The agar tube dilution method was used for the determination of antifungal activity of the methanol extracts of the selected plant species [28]. Samples were prepared by dissolving crude plant extract in DMSO. Culture media was prepared by dissolving 6.5 g of potato dextrose agar per 100 mL distilled water (pH 5.6). Potato dextrose agar (10 mL) was dispensed in screw-capped tubes or cotton-plugged test tubes and autoclaved at 121 °C for 21 min. Tubes were allowed to cool at 50 °C and the potato dextrose agar was loaded with 67 μL of extract pipetted from the stock solutions. The tubes containing the media were then allowed to solidify in slanting position at room temperature. The tubes containing solidified media and plant extract were inoculated with a 4-mm-diameter piece of inoculum taken from a 7 d-old culture of fungus. Controls were run in parallel whereby the respective solvent was used instead of plant extract. The test tubes were incubated at 28 °C for 7 d. Cultures were examined twice weekly during the incubation. Readings were taken by measuring the linear length (mm) of fungus in the slant, and growth inhibition was calculated with reference to negative control. The experiments were performed in triplicate.

Percentage inhibition of fungal growth for each concentration of compound was determined as:

$$ \mathrm{Percentage}\ \mathrm{in}\mathrm{hibition}\ \mathrm{of}\ \mathrm{fungal}\ \mathrm{growth}=100-\frac{\mathrm{Linear}\ \mathrm{growth}\ \mathrm{in}\ \mathrm{test}\ \left(\mathrm{mm}\right)}{\mathrm{Linear}\ \mathrm{growth}\ \mathrm{in}\ \mathrm{control}\ \left(\mathrm{mm}\right)}\times 100 $$

### Cytotoxic brine shrimp assay

Cytotoxic activity of the methanolic plant extracts was tested against brine shrimps hatched in saline solution (known as nauplii) [29]. Methanol extracts of the selected plant species were prepared to make final concentrations of 10, 100 and 1000 mg/mL. An aliquot (2 mL) of each concentration was transferred to a graduated vial, kept for 48 h for solvent evaporation and then dissolved in DMSO before adding the nauplii. Brine shrimp eggs were hatched in a plastic rectangular container that was one-quarter filled with saline solution with general aeration. A plastic separator (with holes) for unequal compartmentation was placed in the container. Eggs were spread into the larger and darker compartment. After 48 h, mature nauplii were collected from the smaller and illuminated side. Ten shrimps were transferred to each vial and saline solution was added to make the final volume 2 mL. Vials were incubated at 25 °C for 24 h, after which the survivors were counted with the aid of a 3× magnifying glass. DMSO and saline solution were used as negative controls and potassium dichromate as the reference standard.

Lethal dose was calculated by linear regression analysis [30].

Abbot’s formula was used to calculate the percentage mortality:

$$ \%\mathrm{Mortality}=\left(\mathrm{Sample}-\mathrm{control}/\mathrm{control}\right)\times 100 $$

### Anti-inflammatory activity

#### Inhibition of protein denaturation method

Inhibition of protein denaturation was determined according to the method of Mizushima et al. [31] with some modifications. The reaction mixture contained the test extract at different concentrations and 1% BSA (aqueous solution). 1 N HCl was used to adjust the pH of the reaction mixture. The samples were heated at 37 °C for 20 min and then 57 °C for 20 min, and allowed to cool. The turbidity of the samples was measured at 660 nm. The experiment was performed in triplicate. Percent inhibition of protein denaturation was calculated as follows:

$$ \mathrm{Percentage}\ \mathrm{inhibition}=\left({\mathrm{A}}_{\mathrm{C}}\ \mathrm{of}\ \mathrm{control}{\textstyle \hbox{--} }{\mathrm{A}}_{\mathrm{C}}\ \mathrm{of}\ \mathrm{test}\ \mathrm{sample}\right)\times 100/{\mathrm{A}}_{\mathrm{C}}\Big) $$

where A_C_ and A_S_ are the absorbance (at 600 nm) of the control and sample, respectively.

#### Human red blood cell (HRBC) membrane stabilization test

Fresh human blood (10 ml) was collected in heparinized centrifuge tubes and centrifuged at 3000 rpm for 10 min and washed 3× with an equal volume of normal saline solution. The volume of the blood was measured and reconstituted as a 10% *v*/v suspension with normal saline [32]. The reaction mixture (2 ml) consisted of 1 ml methanolic plant extract and 1 ml of 10% red blood cell suspension. For the control, saline was added instead of plant extract. Aspirin was used as a standard drug (positive control). The samples were incubated at 56 °C for 30 min, centrifuged at 2500 rpm for 5 min and the absorbance of the supernatant measured at 560 nm. The experiment was performed in triplicate. Percent membrane stabilization activity was calculated by the formula given in Inhibition of protein denaturation method section [33], while the percentage of protection was calculated using the following formula:

$$ \mathrm{Percent}\ \mathrm{of}\ \mathrm{protection}=100-{\mathrm{A}}_{\mathrm{S}}/{\mathrm{A}}_{\mathrm{C}}\times 100 $$

where A_C_ and A_S_ are the absorbance (at 560 nm) of the control and sample, respectively.

### Proteinase inhibitory assay

The proteinase inhibitory assay was performed following the method modified by Oyedepo and Femurewa [34]. The reaction mixture (2 ml) contained 0.06 mg trypsin, 1 ml Tris-HCl buffer (20 mM, pH 7.4) and 1 ml test plant extract sample at different concentrations. The reaction mixture was incubated at 37 °C for 5 min and then 1 ml of 0.8% (*w*/*v*) casein was added. The mixture was incubated for an additional 20 min. Perchloric acid (2 ml of 70%) was added to stop the reaction. The cloudy suspension was centrifuged and the absorbance of the supernatant was measured at 210 nm against Tris-HCl buffer as blank. The experiment was performed in triplicate.

### Statistical analysis

Results were expressed as the mean ± standard error of mean (SEM). The data generated from quantitative assays for phytochemicals and antifungal activity were subjected to ANOVA using Statistix version 8.1. Comparison among mean values was made by Least Significant Difference (LSD) to test significant differences at *P* < 0.05 [35]. Linear regression analysis was used to calculate IC_50_ values. Linear correlations were analyzed by using regression in R software (3.2.2.).

## Results

### Qualitative analysis of phytochemicals, TLC and determination of total phenolic, flavonoid and flavonol contents

The qualitative analysis of phytochemicals revealed the presence of phenolics, flavonoids and terpenoids in all the selected plant extracts. Tannins were observed only in MI*,* AI and CP, whereas phlobatannins (tannins that with hot dilute acids yield a phlobaphene) were found only in CP and TTF. Anthraquinnone was present in significantly higher amounts in TTL followed by AI (Table 2).

**Table 2:** Qualitative analyses of phytochemicals of selected plants

Phytochemicals	*M. indicus* (leaf)	*A. indica* (leaf)	*C. pedicellata* (stem)	*T. terresteris* (fruit)	*T. terresteris* (leaf)
Tannins	+	+	+	−	−
Flavonoids	+++	+++	+++	++	+
Terpenoids	+	+	+	+	+
Phlobatannins	−	−	+	+	−
Anthraquinone	+	++	+	+	+++
Phenolics	++	+	+++	+	+

+++ Strongly positive

++ Moderately positive

+ Weakly positive

- Negative

Results obtained from thin layer chromatography confirmed the presence of different bioactive compounds in the various plant extracts (Table 3). In the leaf extracts of AI, a total of six spots appeared, among which none coincided with the mobility of the standard compounds used. TLC of stem extracts of CP gave a total of nine spots, among which one coincided with salicylic acid, one with *trans*-cinnamic acid and one with caffeic acid. TLC of leaf extracts of MI gave six spots, one of which had a mobility equal to that of quercitin. TLC of TTL gave six spots, among which one coincided with *trans*-cinnamic acid, while TTF gave nine spots, one of which conincided with *trans*-cinnamic acid and one with caffeic acid.

**Table 3:** Thin layer chromatography (TLC) of different parts of methanolic extracts of selected plants

Standards	R*f* value
Caffeic acid	0.85
Quercitin	0.38
Rutin	0.97
*trans*-cinnamic acid	0.74
Salicylic acid	0.6
Plant extracts	R*f* value
*Aristolochia indica*	0.23, 0.26, 0.31, 0.45, 0.56, 0.83
*Cuscuta pedicellata*	0.16, 0.2, 0.25, 0.35, 0.45, 0.61,
	0.75, 0.83, 0.85
*Melilotus indicus*	0.20, 0.25, 0.34, 0.37, 0.56, 0.83
*Tribulus terresteris* (leaf)	0.16, 0.25, 0.30, 0.45, 0.75, 0.83
*Tribulus terresteris* (fruit)	0.16, 0.20, 0.27, 0.35, 0.48, 0.56,
	0.75, 0.83, 0.85

The quantitative analysis revealed that CP exhibited the maximum phenolic, flavonoid and flavonol contents (497, 385 and 139 mg/g, respectively), followed by TTF (426, 371 and 138 mg/g). The minimum phenolic and flavonoid contents were observed in the MI and AI, respectively (Table 4).

**Table 4:** Total phenolics, flavonoid and flavonol content in the dried plant extracts

Plant material	Total phenolic content (mg GAE/g)	Total flavonoid content (mg QE/g)	Total flavonol content (mg QE/g)
*A. indica*	335^d^ ± 5	80^d^ ± 2	72^c^ ± 3
*C. pedicellata* (stem)	497^a^ ± 4	385^a^ ± 8	139^a^ ± 4
*M. indicus*	224^e^ ± 4	116^c^ ± 5	109^b^ ± 4
*T. terrestris* (leaf)	389^c^ ± 4	370^b^ ± 6	55^d^ ± 5
*T. terrestris* (fruit)	426^b^ ± 5	371^b^ ± 8	138^a^ ± 6

Values are means ± SD (*n* = 3). Values in the same column followed by a different letter (a-d) are significantly different (*P* < 0.05)

### Antioxidant activities

**Fig. 1:**
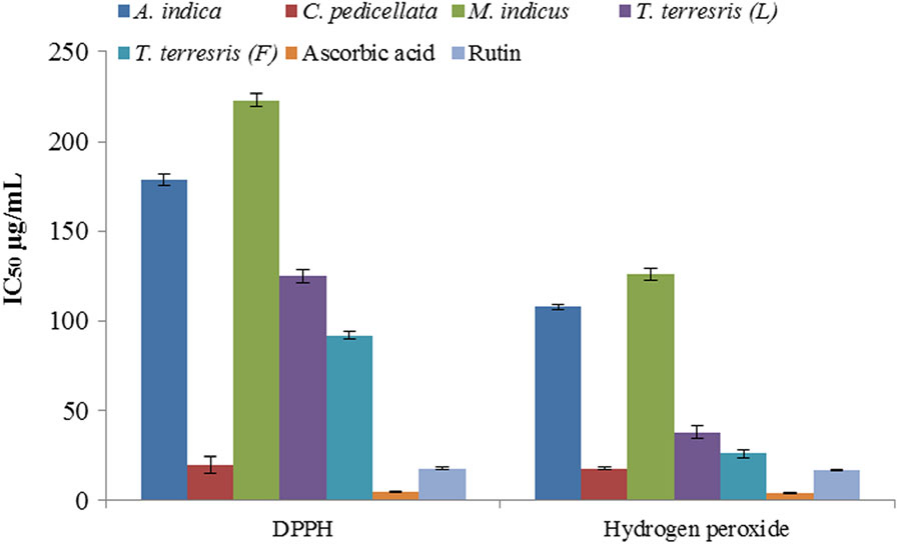
DPPH and H_2_O_2_ scavenging activities of selected plant extracts. Data represent the mean of three replicates

The DPPH assay is commonly used to determine the antioxidant potential of plant extracts/compounds by measuring their ability to act as free radical scavengers. IC_50_ (the substrate concentration that causes 50% loss of DPPH) is used to interpret the assay results. IC_50_ values of scavenging DPPH radicals for CP and TTF extracts were 20 ± 1 and 92 ± 2 μg/mL, respectively; their scavenging ability was found to be lower than that of ascorbic acid and rutin (5 ± 0.4, 18 ± 0.5 μg/mL) (Fig. 1). The DPPH scavenging activity was in the order of CP > TTF > TTL > AI > MI (IC_50_ 20 ± 1, 92 ± 2, 125 ± 3, 179 ± 3, 223 ± 3 μg/mL, respectively).

The IC_50_ values of the selected plant extracts for H_2_O_2_ scavenging activity are presented in Fig. 1. CP and TTF extracts showed strong hydrogen peroxide radical scavenging activity (IC_50_ 18 ± 0.7, 26 ± 2 μg/mL, respectively) whereas the standards ascorbic acid and rutin gave IC_50_ values of 4.5 ± 0.4 and 17 ± 0.6 μg/mL, respectively. The scavenging activity for hydrogen peroxide of selected plant extracts was in the order CP > TTF > TTL > AI > MI.

### Correlation between total phenolic content and IC_50_ values of antioxidants

**Fig. 2:**
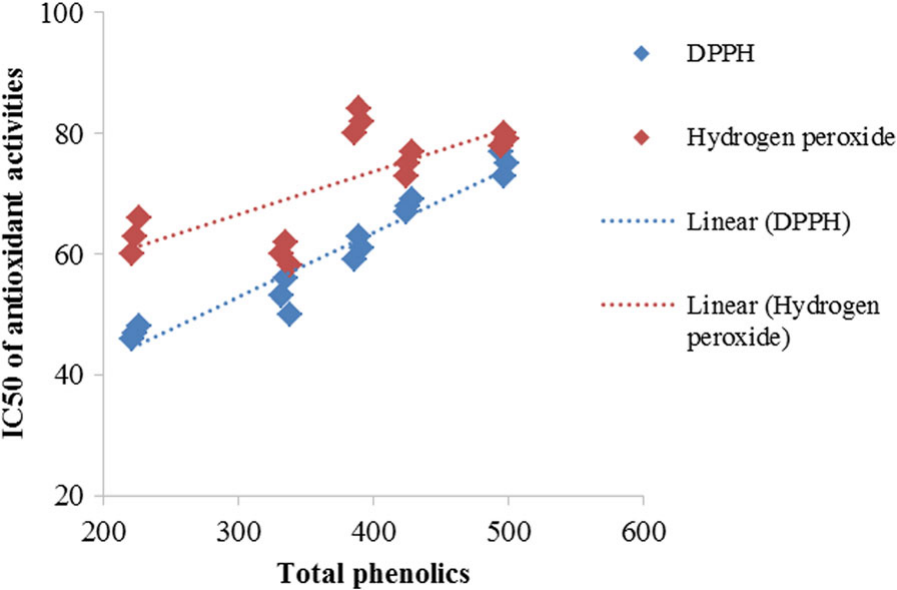
Linear correlation between total phenolic content and IC_50_ values for DPPH and hydrogen peroxide scavenging activities of selected plant extracts, using regression model in R software (3.2.2)

Quantitative analyses of antioxidants and phytochemicals were also used to investigate the correlation between phenolic contents and antioxidant activities (DPPH, H_2_O_2_ radical scavenging activities) in extracts of the selected plant species. The correlation coefficient (R^2^) between total phenolics, DPPH and H_2_O_2_ activities of the five studied plant extracts (Fig. 2) was found to be 0.93.

### Antibacterial activity and determination of MIC and MBC

The plant extracts exhibited considerable antibacterial activity against all the selected strains of microorganisms tested. Results obtained from the agar well diffusion method were used to determine the MIC and MBC (Table 5). The CP extract showed substantial activity against *P. aeruginosa*, *S. aureus*, *K. pneumoneae* and *A. baumannii*, with the lowest MIC values and MBC values, respectively. For activity against *A. baumannii, K. pneumoneae, P. aeruginosa* and *S. aureus*, CP gave the lowest MIC and MBC values, followed by TTF and TTL (Table 5).

**Table 5:** Antibacterial activity determined as zone of inhibition (mm), minimum inhibitory concentration (MIC) and minimum bactericidal concentration (MBC) of selected plant extracts against selected bacterial strains

	Zone of inhibition (mm)							
Plant extracts	*A. baumannii*		*K. pneumoneae*		*P. aeruginosa*		*S. aureus*	
*A. indica*	14 ± 1		13 ± 1.4		16 ± 1.2		15 ± 1.3	
*C. pedicellata*	18 ± 2		20 ± 1.5		23 ± 0.96		22 ± 1.2	
*M. indica*	14 ± 0.9		13 ± 1.7		12 ± 1.1		18 ± 1.3	
*T. terrestris* (leaf)	15 ± 1.2		16 ± 1.2		19 ± 1.3		17 ± 1.1	
*T. terrestris* (fruit)	17 ± 0.9		18 ± 0.85		22 ± 1.1		18 ± 0.86	
Streptomycin	27 ± 1.3		27 ± 0.98		25 ± 1.2		29 ± 1.01	
	*A. baumannii*		*K. pneumoneae*		*P. aeruginosa*		*S. aureus*	
	MIC (μg/mL)	MBC (μg/mL)	MIC (μg/mL)	MBC (μg/mL)	MIC (μg/mL)	MBC (μg/mL)	MIC (μg/mL)	MBC (μg/mL)
*A. indica*	150 ± 4.5	640 ± 10	90 ± 1.5	355 ± 2.2	55 ± 1.21	220 ± 3.5	200 ± 4.3	750 ± 10
*C. pedicellata*	35 ± 1.9	150 ± 6.3	15 ± 1.1	85 ± 1.1	5 ± 0.23	40 ± 1.2	10 ± 1	55 ± 1
*M. indica*	210 ± 7.6	820 ± 11	95 ± 8.5	390 ± 2.3	60 ± 0.9	200 ± 3	70 ± 1.4	250 ± 2.1
*T. terrestris* (leaf)	50 ± 2.1	250 ± 7.8	30 ± 1.1	125 ± 1.4	10 ± 0.57	60 ± 3.5	15 ± 1	80 ± 1.2
*T. terrestris* (fruit)	45 ± 1.9	200 ± 6.5	25 ± 1	100 ± 1.1	8 ± 0.42	50 ± 2.9	12 ± 1	70 ± 1.1
Streptomycin	20 ± 1.2	90 ± 1.8	3 ± 0.4	10 ± 0.5	1 ± 0.11	3 ± 0.3	5 ± 0.9	15 ± 1.4

### Antifungal activity

**Fig. 3:**
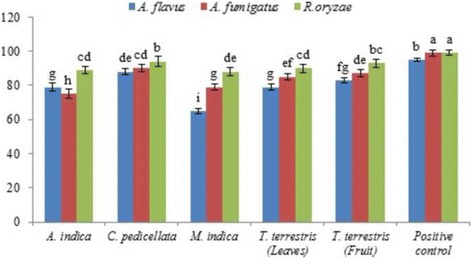
Antifungal potential of methanolic extracts against A: *Aspergillus flavus,* B: *Aspergillus fumigatus,* C: *Rhizopus oryzae,* observed after 7 d of incubation. Data represent the mean of three replicates

Results of the assays for antifungal potential of the selected plant extracts are presented in Fig. 3. Methanolic extracts of CP were highly effective in inhibiting the mycelial growth of *A. flavus* (88% inhibition), followed by TTF and TTL (83% and 79%, respectively). The ranking order for antifungal potential of plant extracts against *A. flavus* was CP > TTF > TTL > MI > AI.

CP extract exhibited the maximum reduction (90%) in the mycelial growth of *A. fumigatus*, followed by TTF and TTL (87% and 85%, respectively). The ranking order for the antifungal potential of plant extracts against *A. fumigatus* was CP > TTF > TTL > MI > AI*.*

CP again was highly effective in inhibiting the mycelial growth (94%) of *R. oryzae*, followed by TTF and TTL (93% and 90%, respectively).

### Correlation between total phenolic content and antimicrobial activities

**Fig. 4:**
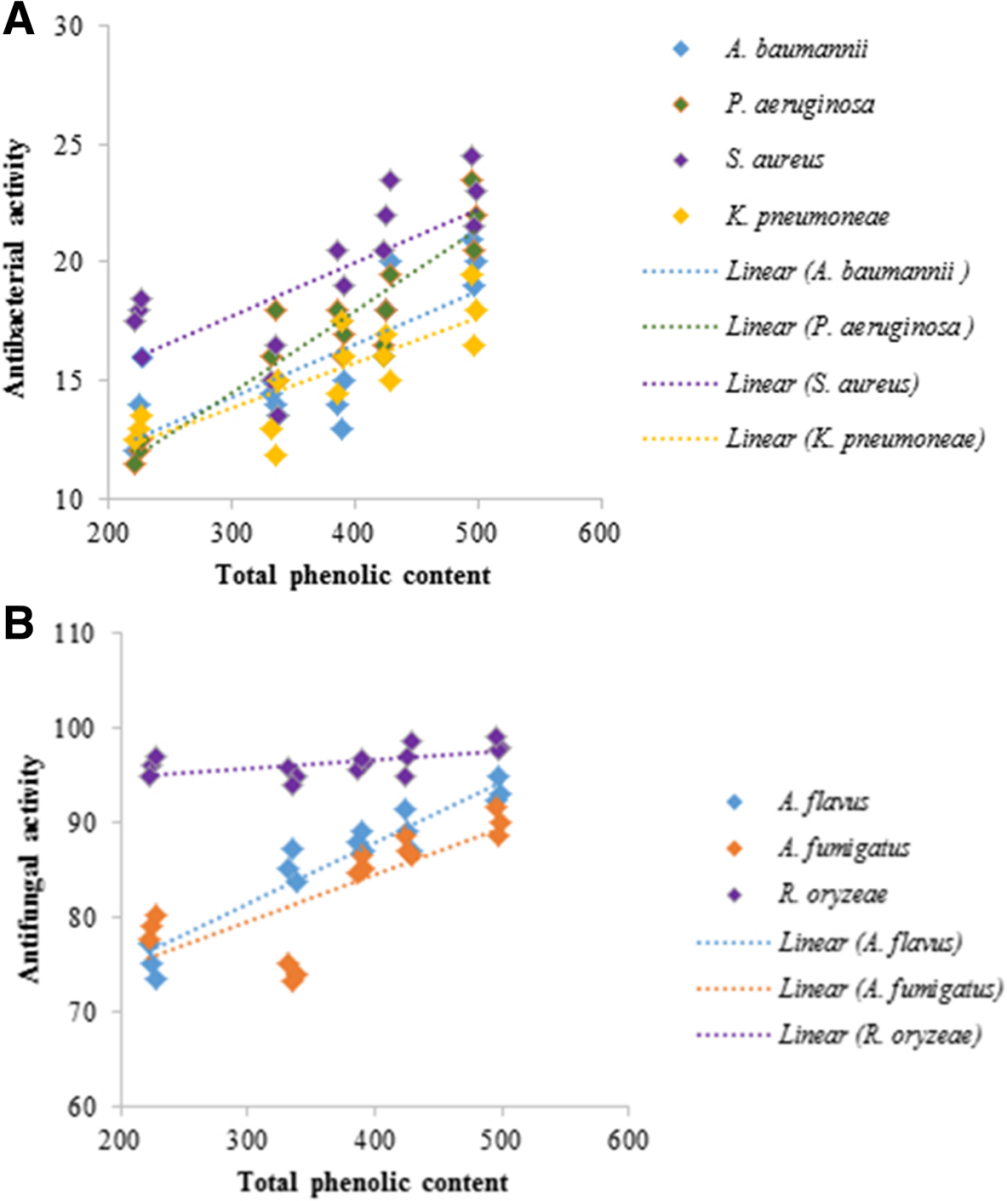
Linear correlation between total phenolic content and (**a**): antibacterial activity and (**b**): antifungal activity of selected plant extracts

Positive correlations were found between the phenolic contents of the selected methanolic plant extracts and inhibition of all of the tested bacteria and fungi (Fig. 4a, b). The correlation coefficients (R^2^) values for activity against *A. baumannii, K. pneumoneae, P. aeruginosa* and *S. aureus* were found to be 0.55, 0.66, 0.87 and 0.46, respectively. The R^2^ values for total phenolic content and antifungal potential against *A. flavus, A. fumigatus* and *R. oryzae* were 0.92, 0.70 and 0.53, respectively.

### Cytotoxic activity

Methanolic plant extracts at three different concentrations (10, 100, 1000 mg/L) were tested for cytotoxicity (Table 6). All the plant extracts showed a cytotoxic effect against brine shrimp cells, with their effectiveness ranked TTF > TTL > AI > MI > CP.

**Table 6:** Percentage mortality of brine shrimps at three different concentrations of plant extracts and respective LD_50_ values

Plant extracts	10 mg/L	100 mg/L	1000 mg/L	LD_50_ mg/L
*A. indica*	1.39	2.48	2.89	66
*C. pedicellata*	1.23	1.55	2.13	109
*M. indicus*	1.09	1.37	2.09	98
*T. terrestris* (leaf)	1.55	2.53	3.56	49
*T. terrestris* (fruit)	2.65	3.12	5.20	38

### Anti-inflammatory and anti-proteinase activity

Test plant extracts (25–200 μg/mL) inhibited albumin denaturation, hemolysis of HRBCs and proteinase activity (Table 7). Albumin denaturation inhibition at the highest concentration of 200 μg/mL was found in CP > TTF > TTL > AI > MI. The IC_50_ of CP was 28 μg/mL.

**Table 7:** Albumin denaturation, membrane protection/stabilization and proteinase inhibition potential of methanol extract of the selected plant species

Test sample			Percent inhibition	
	Conc. (μg/mL)	Albumin denaturation	Membrane protection	Proteinase inhibition
	25	29.7 ± 1.4	26.1 ± 0.9	26 ± 0.8
*A. indica*	50	37.1 ± 1.5	29.4 ± 1.1	28.8 ± 1.1
	100	46.8 ± 1.2	35.7 ± 1.0	35.1 ± 1.1
	200	61.4 ± 1.0	44.6 ± 1.2	43.9 ± 1.5
IC50		129 ± 4.6	247 ± 5.4	256 ± 5.4
Aspirin		84.5 ± 1.1	93.7 ± 1.0	97.1 ± 1.1
	25	43.1 ± 0.8	31.9 ± 0.9	30 ± 1.0
*C. pedicellata*	50	59.5 ± 0.9	37 ± 1.2	34.8 ± 1.3
	100	65.6 ± 1.1	47.6 ± 1.3	42.9 ± 1.3
	200	73.3 ± 1.3	54.5 ± 1.3	52.1 ± 1.5
IC50		28 ± 1.1	151 ± 5.0	175 ± 5.2
Aspirin		91.8 ± 1.1	110.9 ± 1.3	117.1 ± 1.3
	25	17.7 ± 1.5	16.7 ± 1.0	16 ± 1.1
*M. indicus*	50	25.3 ± 1	23.8 ± 1.2	21.9 ± 1.2
	100	33.6 ± 1.5	24.9 ± 1.4	23.9 ± 1.5
	200	48.7 ± 1.5	29.8 ± 1.4	27.6 ± 1.5
IC50		203 ± 5.6	510 ± 6.6	577 ± 7.2
Aspirin		86.2 ± 0.9	95 ± 1.2	105.2 ± 1.3
	25	39.1 ± 1	30.1 ± 1.0	28.5 ± 1.1
*T. terrestris* (fruit)	50	57.6 ± 1.1	45 ± 1.1	43.6 ± 1.2
	100	63.4 ± 1.3	59.7 ± 1.3	59 ± 1.3
	200	71.8 ± 1.5	68.8 ± 1.5	67.9 ± 1.5
IC50		43 ± 1.3	89 ± 2.4	95 ± 2.7
Aspirin		89.2 ± 1.1	95.4 ± 1.2	99.9 ± 1.3
	25	30.7 ± 1.0	28.3 ± 0. 8	27.8 ± 0.9
*T. terrestris* (leaf)	50	38.3 ± 1.1	30.1 ± 1.0	29.4 ± 1.0
	100	48.3 ± 1.1	37.3 ± 1.3	36.4 ± 1.3
	200	63.6 ± 1.3	51.1 ± 1.5	49 ± 1.6
IC50		120 ± 3.9	193 ± 4.8	209 ± 5.6
Aspirin		84.2 ± 1.2	96.8 ± 1.3	99.7 ± 1.4

Values are means ± SD (*n* = 3)

TTF exhibited the maximum inhibition (69%) in the heat-induced hemolysis of HRBCs, followed by CP > TTL. The IC_50_ values for TTF, CP and TTL were 89, 151 and 193 μg/mL, respectively.

The maximum inhibition anti-proteinase activity (68%) was exhibited by TTF at 200 μg/mL, which was followed by CP and TTL. The IC_50_ values for TTF, CP and TTL were 95, 175 and 209 μg/mL, respectively (Table 7).

### Correlation between total phenolic content, anti-inflammatory and anti-proteinase activity

**Fig. 5:**
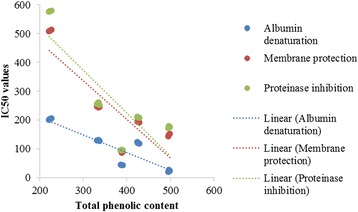
Linear correlation of the total phenolic content versus the anti-inflammatory and anti-proteinase activity of selected plant extracts

The IC50 values for albumin denaturation, membrane protection and proteinase inhibition were calculated (Fig. 5) and used to evaluate correlations with total phenolic content. Positive correlations were found between anti-inflammatory, anti-proteinase activity and total phenolic content. High correlation coefficients (R^2^) values (0.76, 0.72 and 0.996) were found for albumin denaturation, membrane protection and proteinase inhibition, respectively. Therefore, total phenolic content might be used as an indicator in assessing the anti-inflammatory and anti-proteinase activity of the plant extracts tested here.

## Discussion

The medicinal importance of *Aristolochia indica* (AI)*, Cuscuta pedicellata* (CP)*, Melilotus indicus* (MI) and *Tribulus terrestris* (TT) is well appreciated and reported from an ethnobotanical perspective but the biological activities of these plants collected from Chowk Azam, particularly CP, have been insufficiently investigated.

The selected plant extracts contain phenolic, flavonoid, tannin, terpenoid, phlobatannin and anthraquinone compounds. TLC profiling of all selected plant extracts in a chloroform: methanol solvent system also strongly suggested the presence of several bioactive metabolites in these plants. Furthermore, the quantitative analysis of tested plants revealed that methanolic stem extracts of CP*,* TTF and TTL were rich in phenolics and flavonoids. According to Ali et al. [36], *Cuscuta* species contain alkaloids, some glycosides, tannins, flavonoids, steroids and phenolic compounds. We observed the maximum DPPH and H_2_O_2_ [37] radical scavenging activities in the plant extracts of CP, TTF and TTL.

Total phenolic content is considered an important indicator of the antioxidant potential of plant extracts [38]. The correlation coefficient between total phenolic content, DPPH and H_2_O_2_ scavenging activities found here suggests that the phenolic compounds of the selected plant extracts contributed 93% to their antioxidant activities. Similar results of positive correlations between phenolic content and antioxidant activities of several plant extracts have been documented in previous reports [39, 40].

In the present study, the lowest MIC and MBC was observed in CP and found to be highly effective against the selected bacterial pathogens, followed by TTF > TTL extracts. Ali et al. [36] and Faiyyaz et al. [41] have also reported the antimicrobial potential of *C. pedicellata.* The present study also revealed the antifungal potential of plant extracts against *A. flavus, A. fumigatus* and *R. oryzae.* Supporting this data, various medicinal plants are reported to show significant antifungal activity against *A. flavus* [42–44]. The antimicrobial potential of plant extracts can be attributed to the presence of certain bioactive compounds such as phenolics, tannins, flavonoids and polyphenols [45]. Among all these biologically active compounds, Baydar et al. [46] confirmed phenolics as the most significant and active compounds against bacteria as well as fungi. Similarly, the results of antibacterial and antifungal activities obtained in the present study were correlated to their total phenolic contents. Positive correlations between total phenolics of selected plant extracts and their antibacterial as well as antifungal potential were obtained.

Extracts are considered non-toxic if the LD_50_ or LC_50_ is greater than 100 μg/mL in the brine shrimp lethality assay [47]. The mortality percentage and lethal dose (LD_50_) for 50% of the population of nauplii were determined using statistical analysis and a graph of the logarithm of extract dose against lethality percentage [48]. In the present study, the ranking order for cytotoxicity was TTF > TTL > AI > MI > CP. These results are supported by the findings of Menon et al. [49] who reported the strong cytotoxicity of *T. terrestris* fruit extracts. The anti-carcinogenic potential and antitumor activity of *T. terrestris* fruit extracts [47] have also been reported. Our results are also in accord with those of Hossen et al. [50] who demonstrated the moderate cytotoxicity of *Aristolochia indica* methanol extract. According to the previously reported literature, the compounds that show brine shrimp toxicity also tend to have cytotoxic properties against cells of solid tumors found in humans [36].

Protein denaturation and stabilization of human red blood cell membranes were studied to further establish the mechanism of anti-inflammatory action of the traditionally used medicinal plants tested. Inflammation is usually associated with the denaturation of proteins. Results from the present study revealed that CP significantly inhibited protein/albumin denaturation. Methanolic stem extracts of CP had the highest anti-inflammatory potential (strong inhibition of protein denaturation), followed by the TTF and TTL extracts. The selected plant extracts were also effective in stabilizing RBC membranes or inhibiting the heat-induced hemolysis at different concentrations. Chowdhury et al. [51] also reported that methanolic leaf extracts of *Gardenia coronaria* promoted RBC membrane stability. Results from the present study provide evidence for membrane stabilization as an additional mechanism of their anti-inflammatory potential. The potential extracts (CP, TTF, TTL and MI) might inhibit the release of the lysosomal content of neutrophils at inflammation sites but this would need to be investigated. The lysosomal constituents of neutrophils include protease and bactericidal enzymes, which upon extracellular release cause more damage and tissue inflammation [52].

Proteinases have been implicated in arthritic reactions. Neutrophils are reported to be a rich source of serine proteinases, which are localised in lysosomal granules. Leukocyte proteinases are involved in the development of tissue damage during inflammatory reactions and proteinase inhibitors provide substantial protection against this effect [53]. Methanol extracts of *O. corniculata* have been reported to have significant antiproteinase activity [54]. In the present study, high correlation coefficients values were found between total phenolic content and anti-inflammatory as well as anti-proteinase inhibition activity of the selected plant extracts.

## Conclusion

Maximum antioxidant, antimicrobial and anti-inflammatory activities were observed in methanolic CP extracts, which showed strong positive correlations with phenolic content. Results from this study revealed that CP stem extracts and TTF and TTL extracts contain a substantial phenolic content, which was suggested to be the major contributor to their antioxidant, antibacterial, antifungal, anti-inflammatory and anti-proteinase activities. These extracts have potential to be used to prevent food spoilage and to treat inflammation as well as skin irritations. Future research work will be focused on the use of CP stem extracts to protect against peroxidative damage related to carcinogenesis. The effectiveness of extracts of the medicinal plants studied here, particularly of CP, should be further elucidated through additional toxicity and phytochemical analyses to discover effective pharmacological agents.

## Abbreviations

DPPH: 2,2-diphenyl-1-picrylhydrazyl
HRBCs: Human red blood cells
MBC: Minimal bactericidal concentration
MIC: Minimal inhibitory concentration
TLC: Thin layer chromatography
